# Relationship between patent ductus arteriosus and platelet indices in newborn: a systematic review and meta-analysis

**DOI:** 10.3389/fped.2025.1455183

**Published:** 2025-03-21

**Authors:** Yanbing An, Gaowa Arigong, Wuyun Zhao, Rina Su, Xiaoyun Wang

**Affiliations:** ^1^ Department of Neonatology, Inner Mongolia Maternal and Child Health Hospital, Hohhot, China; ^2^ Department of Nursing, Inner Mongolia Maternal and Child Health Hospital, Hohhot, China

**Keywords:** newborn, patent ductus arteriosus, platelet, relationship, meta-analysis

## Abstract

**Background:**

The role of platelet indices in the early hours of life and their potential association with patent ductus arteriosus (PDA) have been subjects of investigation in recent studies. This study aimed to investigate the relationship between PAD and platelet indices in newborn.

**Methods:**

A systematic review and meta-analysis and were preformed based on Chinese databases CNKI and Wanfang database, as well as the international databases PubMed, Web of Science, Cochrane Library, and Embase from their inception to January 31, 2024.

**Results:**

This study included 32 literatures, with 20 of English and 12 of Chinese ones. The meta regression analysis showed that neonates with PDA tend to exhibit lower platelet count (PLT) and plateletcrit (PCT), as well as higher platelet large cell ratio (P-LCR) (all *P* < 0.05), while platelet mass, platelet distribution width (PDW), and mean platelet volume (MPV) remain comparable (all *P* > 0.05).

**Conclusion:**

PDA neonates might have decreased PLT and PCT, while increased P-LCR. These dynamic shifts in platelet indices provide fresh insights into the pathophysiology of PDA and have potential to serve as important indicators for early identification, disease assessment, and personalized treatment decisions in clinical practice.

## Introduction

Patent ductus arteriosus (PDA) is a common cardiovascular condition in preterm newborns, particularly affecting those with gestational age <32 weeks and birth weight <1,500 g (1). Hemodynamically significant PDA (hsPDA) poses challenges in the management of preterm infants and is associated with various complications (2). The role of platelet indices in the early hours of life and their potential association with hsPDA have been subjects of investigation in recent studies (3–5).

While previous research has explored the relationship between platelet parameters and PDA in newborns, there remains ongoing debate regarding the exact nature of this association. Some studies suggest a potential link between platelet activation, as indicated by platelet distribution width (PDW), and the presence of hsPDA in preterm infants (6–8). However, conflicting results have been reported regarding the association of platelet count (PLT), platelet mass, and mean platelet volume (MPV) with hsPDA. To address this discrepancy, several meta-analyses (3, 9, 10) have been conducted to explore the association between PLT and PDA in preterm infants. These meta-analyses have revealed a marginal yet significant association between low PLT in the early days of life and the development of PDA in very preterm infants. However, the precise nature of this association and its implications for clinical practice remain to be fully elucidated.

In this context, our study aims to contribute to the existing literature by conducting a comprehensive meta-analysis of studies investigating the relationship between platelet parameters and hsPDA in premature infants. By synthesizing data from multiple studies, we seek to provide further insights into the potential role of platelets in the pathogenesis of PDA and their utility as biomarkers for identifying infants at risk of developing this condition. Our analysis includes data from a wide range of studies, allowing us to explore the consistency of findings across different cohorts and provide valuable information for future research and clinical decision-making.

## Methods

A protocol was developed prospectively to guide the systematic review and meta-analysis on the association between PDA in newborns and platelet indices. The protocol outlined specific objectives, including identifying relevant studies, determining eligibility criteria for study selection, assessing the quality of included studies, defining clinical outcomes of interest related to hsPDA, and establishing the statistical methodology for data synthesis and analysis. The protocol specified the search strategy to be employed, including the databases to be searched, the keywords and Medical Subject Headings (MeSH) terms to be used, and the inclusion and exclusion criteria for study selection. It also detailed the process for screening and selecting studies, extracting data on platelet parameters and PDA status, and assessing the risk of bias in individual studies.

Furthermore, the protocol outlined the statistical methods to be used for meta-analysis, including the calculation of effect sizes, assessment of heterogeneity between studies, and publication bias analysis. It also described the planned subgroup analyses to explore potential sources of heterogeneity and sensitivity analyses to assess the robustness of the findings. By following this comprehensive protocol, the systematic review and meta-analysis aimed to provide a rigorous and transparent analysis of the association between platelet indices and hsPDA in preterm newborns, contributing valuable insights to the existing literature and informing clinical practice in the management of this common neonatal condition.

### Sources

A thorough literature search was conducted using the Chinese databases CNKI and Wanfang database, as well as the international databases PubMed, Web of Science, Cochrane Library, and Embase from their inception to January 31, 2024. The search strategy involved a combination of keywords related to “patent ductus arteriosus or ductus arteriosus or PDA” and “platelet or platelets or platelet count or platelet counts or thrombocyte or thrombocytopenia” and “neonate or infant” (Supplementary Table S1).

### Study selection

#### Inclusion and exclusion criteria for literatures

Inclusion criteria: (1) The published time of the included literature was within the search deadline; (2) The published articles have been peer-reviewed; (3) The study type was cross-sectional study, including cohort study and case-control study; (4) The subjects were confirmed patients with PDA; (5) A control group was set up in the study, and the association between the untreated ductus arteriosus and platelet indexes could be analyzed through the data reported in the study; (6) Blood samples of the child within 7 days after birth; (7) The language of publication is English or Chinese. Exclusion criteria: (1) The measured platelet index was the result of the measurement after treatment; (2) Repeated data research; (3) suffering from other diseases at the same time; (4) Incomplete or unavailable complete data as well as abstracts, reviews, case reports, letters, and duplicate publications. The related research that has registered in the PROSPERO platform are shown in Supplementary Table S2.

### Data extraction and assessment of study quality

Two investigators (xxx and xxx) independently extracted data on study design, study quality, demographics (Supplementary Table S3), rate of PDA and/or hsPDA, and PLT. A third reviewer (E.V.) checked the data extraction for completeness and accuracy. In cases in which necessary data were missing from the studies, additional information was requested from the authors. Methodological quality was assessed using the Newcastle-Ottawa Scale (NOS) for observational studies (11). This scale uses a star rating system (range: 0–9 stars) scoring three aspects of the study: selection (0–4), comparability (0–2), and outcomes (0–3).

### Statistical analysis

Studies were combined and analyzed using Comprehensive Meta-Analysis v.2.0 software (Biostat Inc., Englewood, N.J., USA). The effect measures estimated were risk ratio (RR) for dichotomous outcomes, and standard mean difference for continuous data. DerSimonian and Laird random-effect models were used to derive random-effect estimates and 95% CI for all outcomes. To identify any study that may have exerted a disproportionate influence on the summary effect, we deleted studies one at a time. Heterogeneity was assessed with the Q statistic and quantified using the *I*^2^ statistic. Because of the small number of studies, a funnel plot analysis to assess publication bias was not conducted. Meta-regression, using random effects (method of moments estimator), was performed to explore the following sources of heterogeneity in the relationship among PDA/hsPDA and PLT, plateletcrit (PCT), platelet large cell ratio (P-LCR), platelet mass, PDW, and MPV. Two-sided *P*-value < 0.05 (0.10 for heterogeneity) was considered statistically significant. The study is reported according to the PRISMA checklist (12).

## Results

This study included 32 literatures, with 20 of English and 12 of Chinese ones. The inclusion and exclusion flowchart for the literatures were shown in Supplementary Figure S1. The main characteristics of the included studies are shown in Supplementary Table S4.

### Comparison of PLT between newborns with PDA and non-PDA

**Figure 1:**
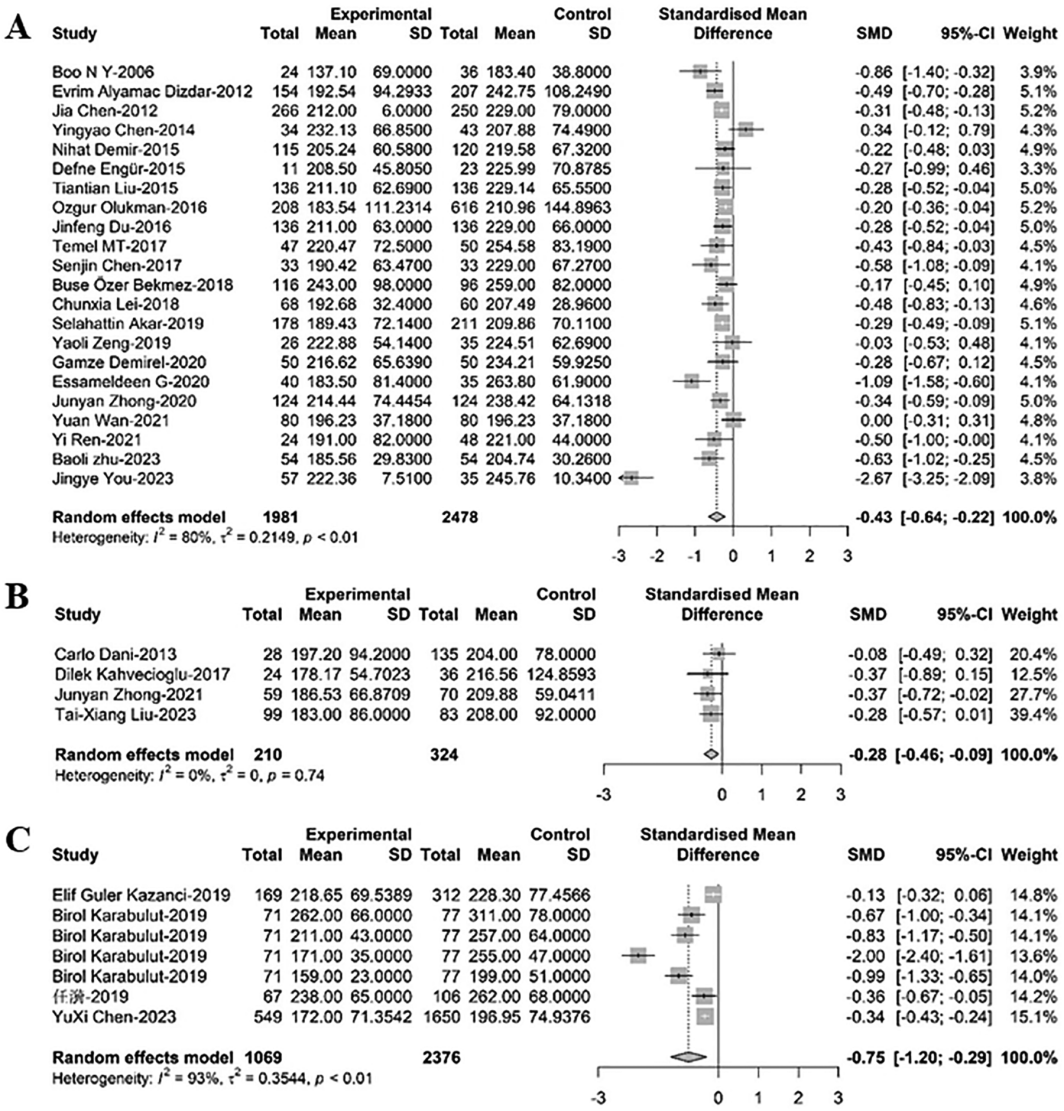
A forest plot for the comparison of PLT between newborns with **(A)** PDA and non-PDA; **(B)** PDA and PDA closure; **(C)** hsPDA and non-hsPDA.

For comparison of PLT between newborns with PDA and those without PDA, 33 relevant studies were included in the meta-regression analysis. Among these, 22 studies comprising 4,459 newborns, with 1,981 diagnosed with PDA, revealed that PLT levels in PDA infants were significantly lower than those in non-PDA infants [SMD = −0.43, 95% CI: (−0.64; −0.22), *P* < 0.001]. Heterogeneity test: *I^2^* = 79.5% [69.7%; 86.2%]; H = 2.21 [1.82; 2.69] (refer to Figure 1 and Supplementary Figure S2A). Four related studies including 534 newborns, with 210 cases of PDA and 324 cases of PDA closure, demonstrated that PLT levels in PDA infants were significantly lower compared to infants with PDA closure [SMD = −0.28, 95%CI: (−0.46; −0.09), *P* = 0.003]. Heterogeneity test: *I^2^* = 0.0% [0.0%; 84.7%]; H = 1.00 [1.00; 2.56] (refer to Figure 1B and Supplementary Figure S2B). Furthermore, seven related studies encompassing 3,445 newborns, with 1,069 cases of hsPDA) and 2,376 non-hsPDA cases, showed that PLT levels in PDA infants were significantly lower than those in non-hsPDA infants [SMD = −0.75, 95%CI: (−1.20; −0.29), *P* = 0.001]. Heterogeneity test: *I^2^* = 93.5% [89.0%; 96.1%]; H = 3.92 [3.02; 5.09] (refer to Figure 1C and Supplementary Figure S2C).

### Comparison of platelet mass between newborns with PDA and non-PDA

**Figure 2:**
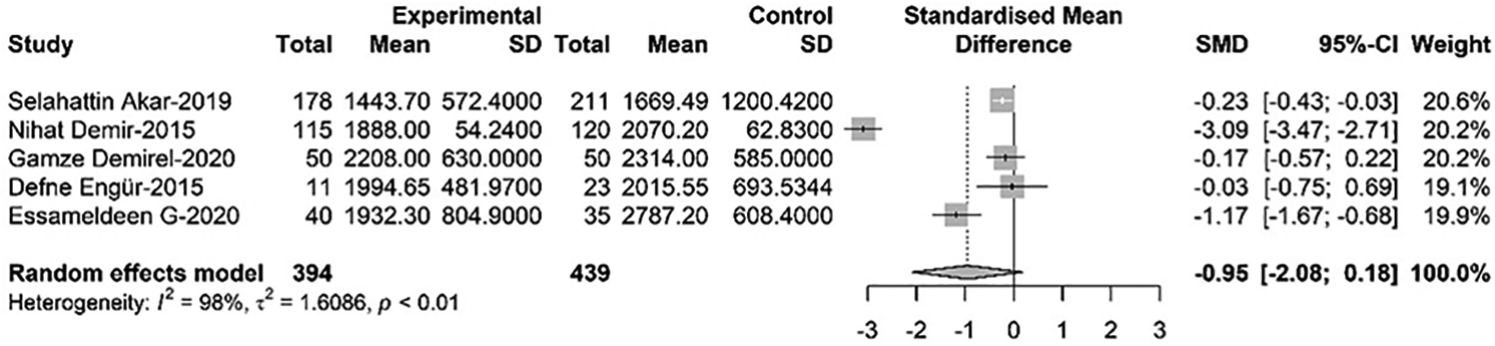
Forest plot for the comparison of platelet quality between newborns with PDA and non-PDA.

Comparing platelet mass between newborns with PDA and non-PDA, five relevant studies were included in the meta-regression analysis. These studies involved a total of 833 newborns, with 394 cases of PDA. Results showed that PDA infants had lower platelet mass than non-PDA infants, although the difference was not statistically significant [SMD = −0.95, 95%CI: (−2.08; −0.18), *P* = 0.100]. Heterogeneity test: *I^2^* = 97.8% [96.6%; 98.6%]; H = 6.82 [5.44; 8.55] (refer to Figure 2 and Supplementary Figure S3).

### Comparison of PDW between newborns with PDA and non-PD

**Figure 3:**
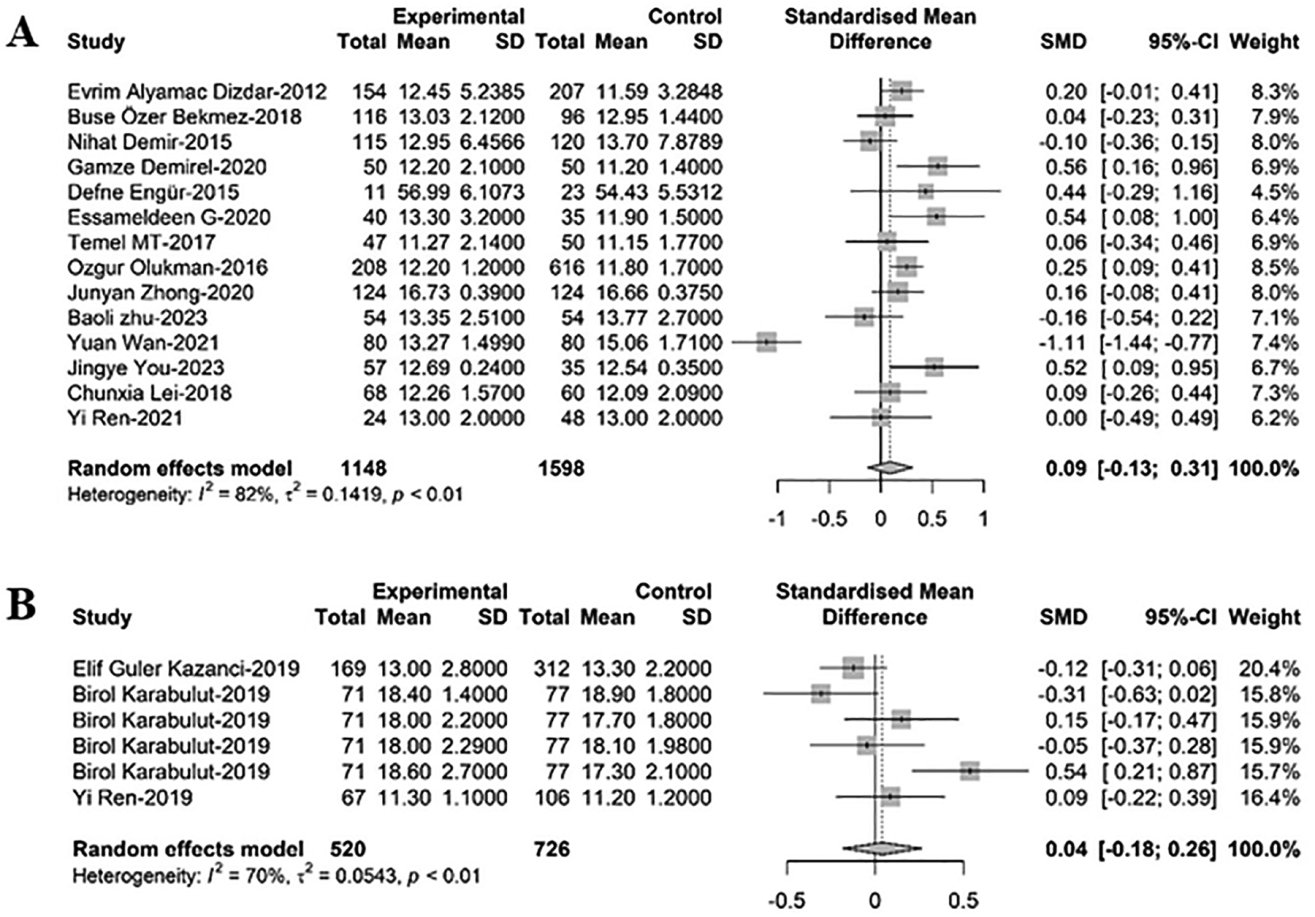
A forest plot for comparison of PDW between newborns with **(A)** PDA and non-PDA; **(B)** hsPDA and non-hsPDA.

To compare PDW between newborns with PDA and non-PDA, 20 relevant studies were included in the meta-regression analysis. Of these, 14 studies with 2,746 newborns, out of which 1,148 had PDA, suggested that PDW levels in PDA infants were higher than those in non-PDA infants, albeit not statistically significant [SMD = 0.09, 95%CI: (−0.13; 0.31), *P* = 0.415]. Heterogeneity test: *I^2^* = 82.2% [71.2%; 88.9%]; H = 2.37 [1.86; 3.01] (refer to Figure 3A and Supplementary Figure S4A). Six additional studies involving 1,246 newborns, with 520 cases of hsPDA and 726 non-hsPDA cases, indicated that PDW levels in PDA infants were higher than in non-hsPDA infants, yet again without statistical significance [SMD = 0.04, 95%CI: (−0.18; 0.26), *P* = 0.719]. Heterogeneity test: *I^2^* = 69.9% [29.3%; 87.1%]; H = 1.82 [1.19; 2.79] (refer to Figure 3B and Supplementary Figure S4B).

### Comparison of MPV between newborns with PDA and non-PD

**Figure 4:**
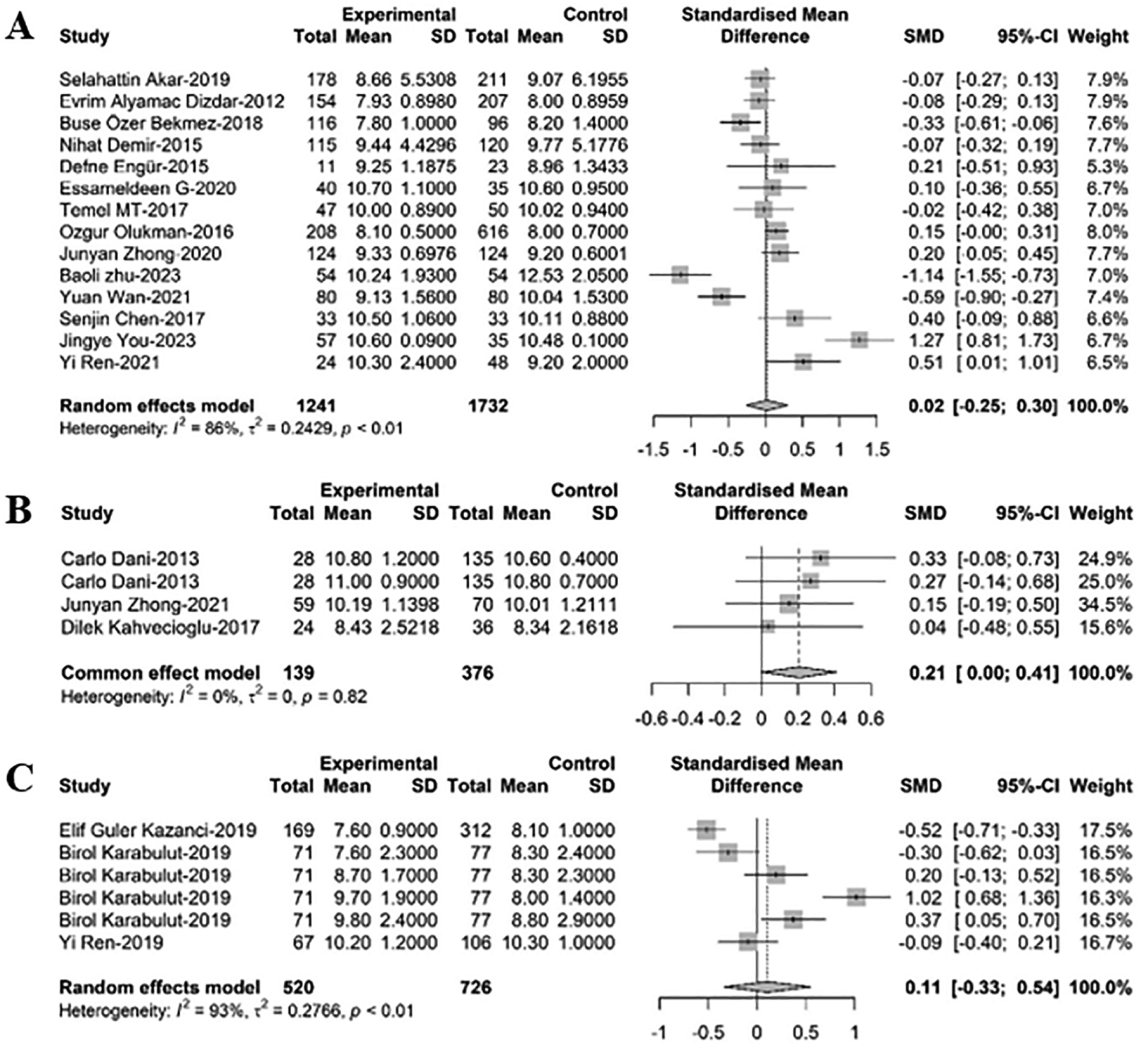
A forest plot for comparison of MPV between newborns with **(A)** PDA and non-PDA; **(B)** PDA and non-PDA; **(C)** hsPDA and non-hsPDA.

In comparing MPV between newborns with PDA and non-PDA, 23 relevant studies were analyzed via meta-regression. Among these, 14 studies included 2,973 newborns, with 1,241 having PDA. MPV levels in PDA infants were found to be greater than those in non-PDA infants, though the difference lacked statistical significance [SMD = 0.02, 95%CI: (−0.25; 0.30), *P* = 0.870]. Heterogeneity test: *I^2^* = 85.9% [78.0%; 91.0%]; H = 2.67 [2.13; 3.33] (refer to Figure 4 and Supplementary Figure S5A). Three studies with 515 newborns, 139 of whom had PDA and 376 had PDA closure, showed that MPV levels in PDA infants were slightly greater than in infants with PDA closure, but this difference was not statistically significant [SMD = 0.21, 95%CI: (0.00; 0.41), *P* = 0.82]. Heterogeneity test: *I^2^* = 0.0% [0.0%; 89.6%]; H = 1.00 [1.00; 3.10] (refer to Figure 4B and Supplementary Figure S5B). Another six studies including 1,246 newborns, with 520 cases of hsPDA and 726 non-hsPDA cases, revealed that MPV levels in PDA infants were marginally greater than in non-hsPDA infants, yet this difference did not reach statistical significance [SMD = 0.11, 95%CI: (−0.33; 0.54), *P* = 0.638]. Heterogeneity test: *I^2^* = 93.0% [87.5%; 96.1%]; H = 3.78 [2.83; 5.06] (refer to Figure 4C and Supplementary Figure S5C).

### Comparison of PCT between newborns with PDA and non-PDA

**Figure 5:**
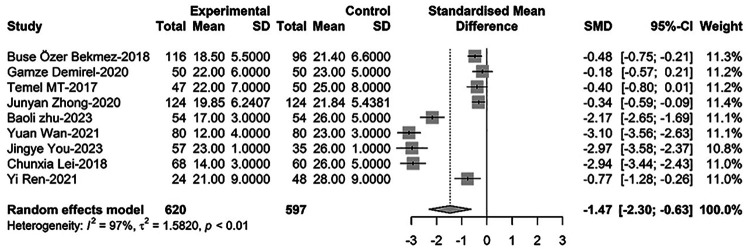
Forest plot for comparison of PCT between newborns with PDA and non-PDA.

Regarding PCT comparison between newborns with PDA and non-PDA, nine relevant studies were incorporated in the meta-regression analysis. These studies collectively included 1,217 newborns, with 620 diagnosed with PDA. The findings indicated that PCT levels in PDA infants were significantly lower than those in non-PDA infants [SMD = −1.47, 95%CI: (−2.30; −0.63), *P* = 0.001]. Heterogeneity test: *I^2^* = 97.0% [95.7%; 97.9%]; H = 5.77 [4.82; 6.90] (refer to Figure 5 and Supplementary Figure S6).

### Comparison of P-LCR between newborns with PDA and non-PDA

**Figure 6:**
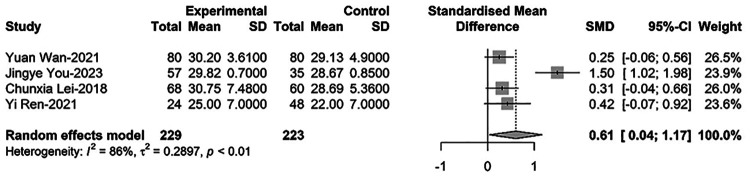
Forest plot for comparison of P-LCR between newborns with PDA and non-PDA.

Finally, for comparing P-LCR between newborns with PDA and non-PDA, four relevant studies were included in the meta-regression analysis. These studies involved 452 newborns, with 229 cases of PDA. The results showed that P-LCR levels in PDA infants were significantly higher than those in non-PDA infants [SMD = 0.61, 95%CI: (0.04; 1.17), *P* = 0.036]. Heterogeneity test: *I^2^* = 85.5% [64.4%; 94.1%]; H = 2.63 [1.68; 4.13] (refer to Figure 6 and Supplementary Figure S7).

## Discussion

This study demonstrates that neonates with PDA tend to exhibit lower PLT and PCT, as well as higher P-LCR, while Platelet Mass, PDW, and MPV remain comparable. These alterations in platelet indices not only provide novel insights into the pathophysiology of PDA but also furnish new leads for early diagnosis, disease evaluation, and individualized therapeutic strategies in clinical practice.

Consistent with previous research (3, 13, 14), this study confirms that PDA-afflicted neonates present with decreased PLT and PCT, likely due to the pivotal role platelets play in hemostasis and thrombosis within the cardiovascular system (15, 16). The presence of an open ductus arteriosus may result in abnormal circulation, particularly when shunting occurs between pulmonary and systemic circulations, possibly leading to increased platelet consumption and hence depletion of circulating platelets (17). The decline in PCT may reflect impaired functional capacity of platelets in the closure process of the arterial duct, or over-mobilization causing relative insufficiency locally or systemically. Moreover, altered hemodynamics in infants with PDA could exacerbate shear stress on platelets, accelerating their apoptosis or destruction (18, 19).

Additionally, our findings reveal elevated P-LCR in PDA-positive neonates, suggesting morphological and functional changes in platelets within this population. An increased P-LCR may denote heightened platelet activation status or accelerated platelet turnover in response to abnormal pressure and flow dynamics within the cardiovascular system (20). Alternatively, it might be associated with inflammatory responses, as previous studies have implicated prenatal infections and upregulated inflammatory factors in the etiology of PDA (21), which can influence both platelet production and clearance mechanisms, ultimately contributing to variations in platelet parameters. Furthermore, no significant differences were observed in platelet mass, PDW, and MPV between neonates with and without PDA. This implies that, despite changes in platelet numbers and certain ratios, fundamental quality attributes like PDW and MPV do not show marked discrepancies, suggesting that although the total PLT and certain kinetic parameters are affected, the basic structure and maturation state of platelets are unlikely to be significantly altered due to the presence of PDA.

In addition, considering the diminutive size of platelets, accurate measurement is critical for reliable interpretation of platelet indices in neonates with PDA. PLT and derived indices such as PCT, P-LCR, PDW, and MPV are typically obtained via automated hematology analyzers that use multi-angle laser light scattering or impedance methods to enumerate and characterize these cellular fragments (22). The accuracy of platelet measurements can be influenced by several factors, including pre-analytical variables like sample handling, anticoagulant choice, and timing of blood collection relative to feeding or other interventions (23). Analytical challenges may also arise from the presence of platelet clumping or microclots, which can lead to underestimation of PLT counts and affect derived parameters (24). To mitigate these issues, rigorous quality control measures should be implemented, encompassing standardized protocols for specimen collection and processing, calibration of laboratory equipment, and proficiency testing. Additionally, laboratories should adhere to guidelines set forth by regulatory bodies and professional organizations to ensure optimal performance and reliability of platelet-related assays. In this meta-analysis, we have attempted to account for these variables by including studies that report adherence to strict quality assurance practices and by performing sensitivity analyses to evaluate the robustness of our findings. Future research endeavors should aim to standardize methodologies across studies to further enhance the comparability and reproducibility of platelet index data in neonatal populations.

Moreover, the observed alterations in platelet indices (lower PLT and PCT, elevated P-LCR) suggest their potential utility as adjunct biomarkers for early PDA identification. In clinical settings, echocardiography remains the gold standard for PDA diagnosis, but its accessibility may be limited in resource-constrained regions or in cases requiring rapid screening. Platelet indices, routinely measured in neonatal blood tests, could serve as a cost-effective and readily available triage tool. For example, neonates with thrombocytopenia (low PLT) or elevated P-LCR might be prioritized for echocardiographic evaluation, thereby reducing diagnostic delays. This approach aligns with recent studies advocating for the integration of hematological parameters into neonatal PDA risk stratification models (8). In addition, the dynamic changes in platelet indices may inform clinical decisions regarding PDA management. Hemodynamically significant PDA (hsPDA) often necessitates pharmacological or surgical intervention, but current criteria for defining hsPDA lack consensus. Our findings imply that platelet indices could complement echocardiographic and clinical parameters in risk assessment. Specifically, (1) risk stratification: Lower PLT and PCT may reflect platelet consumption linked to PDA-related inflammation, potentially indicating disease severity. Neonates with severe thrombocytopenia might benefit from earlier intervention. (2) monitoring therapeutic response: Serial measurement of platelet indices post-treatment (e.g., after ibuprofen administration) could help evaluate treatment efficacy. A rebound in PLT or normalization of P-LCR might correlate with ductal closure, offering a quantifiable marker for clinicians. However, these still require a large amount of clinical data for further exploration and validation.

In this study, which exclusively relies on a meta-analysis of observational studies, several limitations must be acknowledged. Firstly, limitations on causal inference. Due to the reliance solely on observational studies, our research cannot definitively establish a causal relationship between PDA in newborns and platelet indices. Observational designs inherently restrict us to describing associations rather than causality, potentially influenced by unmeasured or unknown confounding factors. Secondly, heterogeneity across studies. Despite rigorous screening and meta-analytic procedures, considerable heterogeneity might exist among the included studies with respect to participant characteristics (e.g., gestational age, birth weight, maternal health status), diagnostic criteria for PDA, methods of platelet index measurement, and timepoints of assessment, all of which may compromise the reliability and generalizability of the meta-analysis results. Thirdly, insufficient sample sizes and statistical power. For certain platelet indicators within secondary outcomes or subsets of high-quality studies, smaller sample sizes may lead to unstable effect estimates and reduced statistical power, thereby limiting our comprehensive understanding of the association between PDA and these specific platelet measures. Finally, this study included only articles published in English and Chinese, which may not fully reflect the current body of evidence. Expanding the scope to incorporate research published in other languages would provide a more comprehensive representation of global findings and strengthen the robustness of our conclusions.

In conclusion, this study revealed that, in PDA neonates, decreased PLT and PCT might occur, as well as increased P-LCR. These dynamic shifts in platelet indices provide fresh insights into the pathophysiology of PDA and have potential to serve as important indicators for early identification, disease assessment, and personalized treatment decisions in clinical practice. Looking forward, future research should delve deeper into the specific mechanisms by which platelets are involved in the development of PDA, particularly focusing on the reasons behind increased platelet consumption, functional alterations, and morphological changes. Moreover, observations regarding platelet parameters in PDA newborns can guide the development of more precise early screening methods and individualized intervention strategies.

## Data Availability

The original contributions presented in the study are included in the article/Supplementary Material, further inquiries can be directed to the corresponding author.

## References

[1] Hamrick SEG Sallmon H Rose AT Porras D Shelton EL Reese J Patent ductus arteriosus of the preterm infant. Pediatrics. (2020) 146(5):e20201209. 10.1542/peds.2020-1209 33093140 PMC7605084

[2] Santos J Soares P Ferreras C Flor-de-Lima F Guimarães H. Patent ductus arteriosus in preterm newborns: a tertiary hospital experience. Rev Port Cardiol. (2022) 41(2):109–18. 10.1016/j.repc.2021.01.008 33934914

[3] Ding R Zhang Q Duan Y Wang D Sun Q Shan R. The relationship between platelet indices and patent ductus arteriosus in preterm infants: a systematic review and meta-analysis. Eur J Pediatr. (2021) 180(3):699–708. 10.1007/s00431-020-03802-5 32949292

[4] Ali Engür M Engür D. Platelet-rich plasma for patent ductus arteriosus: an orthopaedic surgeon’s perspective. Cardiol Young. (2014) 24(3):385–7. 10.1017/s1047951114000146 24552664

[5] Ren Y Gao XY Wang HY Yang B Zhao DD Huang D [Predictive value of platelet aggregation rate in hemodynamically significant patent ductus arteriosus in preterm infants]. Zhonghua Er Ke Za Zhi. (2021) 59(2):113–8. 10.3760/cma.j.cn112140-20200818-00807 33548957

[6] Ohlsson A Shah PS. Paracetamol (Acetaminophen) for patent ductus arteriosus in preterm or low birth weight infants. Cochrane Database Syst Rev. (2018) 4(4):Cd010061. 10.1002/14651858.CD010061.pub3 conflict of interest to declare. 29624206 PMC6494526

[7] Ohlsson A Shah PS. Paracetamol (Acetaminophen) for patent ductus arteriosus in preterm or low birth weight infants. Cochrane Database Syst Rev. (2020) 1(1):Cd010061. 10.1002/14651858.CD010061.pub4 conflict of interest to declare. 31985831 PMC6984659

[8] Özer Bekmez B Tayman C Büyüktiryaki M Çetinkaya AK Çakır U Derme T. A promising, novel index in the diagnosis and follow-up of patent ductus arteriosus: red cell distribution width-to-platelet ratio. J Clin Lab Anal. (2018) 32(9):e22616. 10.1002/jcla.22616 29978492 PMC6817018

[9] Simon SR van Zogchel L Bas-Suárez MP Cavallaro G Clyman RI Villamor E. Platelet counts and patent ductus arteriosus in preterm infants: a systematic review and meta-analysis. Neonatology. (2015) 108(2):143–51. 10.1159/000431281 26159239

[10] Mitra S Chan AK Paes BA. The association of platelets with failed patent ductus arteriosus closure after a primary course of indomethacin or ibuprofen: a systematic review and meta-analysis. J Matern Fetal Neonatal Med. (2017) 30(2):127–33. 10.3109/14767058.2016.1163684 26955892

[11] Lo CK Mertz D Loeb M. Newcastle-Ottawa Scale: comparing reviewers’ to authors’ assessments. BMC Med Res Methodol. (2014) 14:45. 10.1186/1471-2288-14-45 24690082 PMC4021422

[12] Page MJ McKenzie JE Bossuyt PM Boutron I Hoffmann TC Mulrow CD The PRISMA 2020 statement: an updated guideline for reporting systematic reviews. Br Med J. (2021) 372:n71. 10.1136/bmj.n71 33782057 PMC8005924

[13] Kahvecioglu D Erdeve O Akduman H Ucar T Alan S Çakır U Influence of platelet count, platelet mass index, and platelet function on the spontaneous closure of ductus arteriosus in the prematurity. Pediatr Neonatol. (2018) 59(1):53–7. 10.1016/j.pedneo.2017.01.006 28739214

[14] Demir N Peker E Ece İ Ağengin K Bulan KA Tuncer O. Is platelet mass a more significant indicator than platelet count of closure of patent ductus arteriosus? J Matern Fetal Neonatal Med. (2016) 29(12):1915–8. 10.3109/14767058.2015.1067296 26169703

[15] Reiss AB Grossfeld D Kasselman LJ Renna HA Vernice NA Drewes W Adenosine and the cardiovascular system. Am J Cardiovasc Drugs. (2019) 19(5):449–64. 10.1007/s40256-019-00345-5 30972618 PMC6773474

[16] Lee C Li X. Platelet-derived growth factor-C and -D in the cardiovascular system and diseases. Mol Aspects Med. (2018) 62:12–21. 10.1016/j.mam.2017.09.005 28965749

[17] Sallmon H Delaney CA. Platelets and ductus arteriosus closure in neonates. Semin Perinatol. (2023) 47(2):151719. 10.1016/j.semperi.2023.151719 36925318

[18] Akpinar I Sayin MR Gursoy YC Karabag T Kucuk E Buyukuysal MC Plateletcrit. A platelet marker associated with saphenous vein graft disease. Herz. (2014) 39(1):142–8. 10.1007/s00059-013-3798-y 23575980

[19] Cerit L Cerit Z. Relationship between coronary tortuosity and plateletcrit coronary tortuosity and plateletcrit. Cardiovasc J Afr. (2017) 28(6):385–8. 10.5830/cvja-2017-023 28470327 PMC5885048

[20] Verdoia M Pergolini P Rolla R Nardin M Barbieri L Schaffer A Platelet larger cell ratio and high-on treatment platelet reactivity during dual antiplatelet therapy. Cardiovasc Drugs Ther. (2015) 29(5):443–50. 10.1007/s10557-015-6616-3 26428927

[21] Wei YJ Hsu R Lin YC Wong TW Kan CD Wang JN. The association of patent ductus arteriosus with inflammation: a narrative review of the role of inflammatory biomarkers and treatment strategy in premature infants. Int J Mol Sci. (2022) 23(22):13877. 10.3390/ijms232213877 36430355 PMC9699120

[22] Prompetchara E Parnsamut C Chirapanuruk A Ketloy C. Performance evaluation of a novel platelet count parameter, hybrid platelet count, on the BC-780 automated hematology analyzer. Clin Chem Lab Med. (2023) 62(4):690–7. 10.1515/cclm-2023-1000 37855253

[23] Schubert P Culibrk B Coupland D Levin E Devine DV. Impact of sample volume and handling time during analysis on the *in vitro* quality measurements of platelet concentrates held in syringes. Int J Lab Hematol. (2011) 33(6):579–85. 10.1111/j.1751-553X.2011.01327.x 21545688

[24] Woo S Kim B Heo NH Kim MS Yoon YA Choi YJ. Reliability of the DI-60 digital image analyzer for detecting platelet clumping and obtaining accurate platelet counts. Ann Lab Med. (2024) 44(6):572–5. 10.3343/alm.2024.0003 38639011 PMC11375185

