# Supplementary material for: Relationship between patent ductus arteriosus and platelet indices in newborn: a systematic review and meta-analysis
Source: Front Pediatr. 2025 Mar 21;13:1455183. doi: 10.3389/fped.2025.1455183 (PMC11968669; doi:10.3389/fped.2025.1455183)
Supplement: Supplementary file 1 [file Datasheet1.pdf]

Supplementary Tables

Table S1 Search strategy

Database	Search strategy	Results
Chinese databases CNKI	(patent ductus arteriosus OR ductus arteriosus OR PDA) AND (platelet OR platelets OR platelet count OR plateletcounts OR thrombocyte OR thrombocytopenia) AND (neonate OR infant)	22
Wanfang database		107
PubMed		188
Web of Science		157
Cochrane Library		56
Embase		603

Table S2 The related research that have registered in the PROSPERO platform

Record	XAC	AUT	TTL	XYR
1	CRD42019146012	Peter Evans, Deirdre O'Reilly, Jonathan Flyer, Souvik Mitra, Roger Soll	Indomethacin for symptomatic patent ductus arteriosus in preterm infants [Cochrane protocol]	2019
2	CRD42021279329	Maria Pierro, Joanna Ulan-Drozdowska, Joanna Seliga-Siwecka, Marta Szumska, Klaudia Krasuska, Niazy Al-Assaf, Margit Benke, Stefano Ghirardello, Roberto Chioma, Krzysztof Włodarczyk, Antonio Spagarino, Roy Philip	Platelet transfusion and risk of bronchopulmonary dysplasia: a systematic review and meta-analysis	2021
3	CRD42019146061	Vishal Kapoor, Manoj Malviya, Roger Soll	Lipid emulsions for parenterally fed preterm infants [Cochrane protocol]	2019
4	CRD42019140850	Laurens Eeftinck Schattenkerk, Taco Boltje	Evaluation of histological findings associated with spontaneous intestinal perforation vs other causes of perforation in the neonatal period	2019
5	CRD42021279564	Maria Pierro, Joanna Ulan-Drozdowska, Joanna Seliga-Siwecka, Marta Szumska, Klaudia Krasuska, Krzysztof Włodarczyk, Stefano Ghirardello, Antonio Spagarino, Margit Benke, Niazy Alassaf, Roy Philip, Roberto Chioma	Fresh Frozen Plasma transfusion and risk of Bronchopulmonary Dysplasia: a Systematic review and Meta-analysis	2021

Table S3 Literature quality evaluation by Newcastle-Ottawa scale

Author	1) Representativeness of the exposed cohort	2) Selection of the non exposed cohort	3) Ascertainment of exposure	4) Demonstration that outcome of interest was not present at start of study	1) Comparability of cohorts on the basis of the design or analysis	2) study controls for any additional factor	1) Assessment of outcome	2) Was follow-up long enough for outcomes to occur	3) Adequacy of follow up of cohorts
Yu-Xi Chen	1	1	1	0	1	1	1	1	0
Mehmet KÜÇÜK	1	1	1	0	1	1	1	1	0
Selahattin Akar	1	1	1	0	1	1	1	0	0
Evrim Alyamac Dizdar	1	1	1	0	1	1	1	0	0
Maria Pilar Bas-Suárez	1	1	1	0	1	1	1	0	0
Buse Özer Bekmez	1	1	1	0	1	1	1	0	0
Boo N Y	1	1	1	1	1	1	1	1	1
Ying-Yao Chen	1	1	1	0	1	1	1	0	0
Carlo Dani	1	1	1	0	1	1	1	0	0
Nihat Demir	1	1	1	0	1	1	1	0	0
Gamze Demirel	1	1	1	0	1	1	1	0	0
Defne Engür	1	0.5	1	0	1	1	1	1	1
Essameldeen G	1	1	1	0	1	1	1	0	0
Elif Guler Kazanci	1	1	1	1	1	1	1	0	0
Temel MT	1	1	1	1	1	1	1	0	0
Junyan Zhong	1	1	1	1	1	1	1	0	0
Ozgur Olukman	1	1	1	1	1	1	1	0	0
Tai-Xiang Liu	1	1	1	1	1	1	1	0	0
Birol Karabulut	1	1	1	1	1	1	1	0	0
Dilek Kahvecioglu	1	1	1	0	1	1	1	0	0

Yanjun Z	1	1	1	1	1	1	1	0	0
Baoli Z	1	1	1	1	1	1	1	0	0
Yuan W	1	1	1	1	1	1	1	0	0
Jia C	1	1	1	1	1	1	1	0	0
Tiantian L	1	1	1	1	1	1	1	0	0
Senjing C	1	1	1	1	1	1	1	0	0
Jingye Y	1	1	1	1	1	1	1	0	0
Chunxia L	1	1	1	1	1	1	1	0	0
Yali Z	1	1	1	1	1	1	1	0	0
Jingfeng D	1	1	1	1	1	1	1	0	0
Yi R	1	1	1	1	1	1	1	0	0
Yi R	1	1	1	1	1	1	1	0	0

Table S4 The main characteristics of the included studies.

Author	Publication time	Study design	Sample size	Day PDA assessment	PLT	Platelet mass	Platelet count nadir	RPR	PDGF	MPR	PDW	MPV	PCT	P-LCR
Yu-Xi Chen	2023	Retrospective cohort study	sPDA group, n=549；nsPDA group, n=1650	3 day	172.0 (124.0, 220.0); 198.0 (146.0, 247.0)									
Mehmet KÜÇÜK	2018	Retrospective study	sPDA group, n=46；Spontaneous Closure of PDA group, n=29	1day; 3day	1day: 218.0 ± 76.0, 222.5 ± 68.0; 3day 187.9 ± 102.7, 209.9 ± 81.5							1day: 8.55 ± 0.6, 8.6 ± 0.7; 3day 9.03 ± 0.8, 8.84 ± 0.8		
Selahattin Akar	2019	Retrospective study	Case group, n=178；Control group, n=211	1 day	189.43 ± 72.14; 209.86 ± 70.11	1443.70 ± 572.40; 1669.49 ± 1200.42						7.4(5.5–12.9); 7.7(5.5–13.8)		
Evrim Alyamac Dizdar	2012	Retrospective study	Case group, n=154；Control group, n=207	3 day	182（134-260）; 241(171-316)						12.1 (9.1–16.1); 11.8 (9.3–13.7)	8.0 (7.3–8.5); 8.0(7.4–8.6)		

Maria Pilar Bas-Suárez	2014	Retrospective study	sPDA group, n=105；nsPDA group, n=89	1-2 day			221 (175 – 263); 213 (176 – 261)							
Buse Özer Bekmez	2018	Retrospective cohort study	Case group, n=116；Control group, n=96	7 day	243 ± 98; 259 ± 82			0.084 ± 0.069 ; 0.068 ± 0.024			13.03 ± 2.12; 12.95 ± 1.44	7.8 ± 1.0; 8.2 ± 1.4	0.185 ± 0.055；0.214 ± 0.066	
Boo N Y	2006	Prospective observational study	Case group, n=24；Control group, n=36	7 day	137.1 (SD：69.0); 183.4 (SD：38.8)									
Ying-Yao Chen	2014	Retrospective cohort study	Case group, n=34；Control group, n=43	7 day	232.13±66.85; 207.88±74.49									
Carlo Dani	2013	Retrospective study	PDA group, n=135；Spontaneous Closure of PDA group,	0-1day; 1-2day	PDA group 0-1day:204.0 ±78.0，1-2day：191.2 ±116.1;							PDA group 0-1day:10.8 ± 1.2，1-2day：11.0 ± 0.9;		

			n=28		Spontaneous Closure of PDA group 0-1day:197.2±94.2，1-2day：160.8 ± 70.8							Spontaneous Closure of PDA group 0-1day:10.6 ± 0.4，1-2day：10.8 ± 0.7		
Nihat Demir	2015	Retrospective study	Case group, n=115；Control group, n=120	3 day	205.24±60.58；219.58±67.32	1888±54.24; 2070.2±62.83					11.4 (9.3-17.9); 11.2 (9.5-20)	9.4 (6.5-12.4); 9.1 (6.6-13.5)		
Gamze Demirel	2020	Case-control study	Case group, n=50；Control group, n=50	1 day	216.620± 65.639; 234.210±59.925	2208 ± 630; 2314 ± 585					12.2 ± 2.1; 11.2 ± 1.4		0.22 ± 0.06; 0.23 ± 0.05	
Defne Engür	2015	Prospective observational study	Case group, n=11；Control group, n=23	1 day	208.5 (181.5-235.5); 220 (183.5-273.2)	1998.7 (1709.1-2277.3); 1936 (1608.1-2485.8)			Day 2:651.69(438.71-760.9); 899.5(493.99-1072.84); day 5：874.69(704.50-909.38); 1099.6(754-1273.99)		56.4% (53.6-60.8); 54% (51.1-58.1)	9.1(8.6-10); 9(8.1-9.8)		

Essameldeen G	2020	Prospective observational study	Case group, n=40；Control group, n=35	3 day	183.5 (81.4); 263.8 (61.9)	1932.3 (804.9); 2787.2 (608.4)			day 2 ng/dl: 1.45(0.53); 1.71(0.69); day 5: 1.65(0.81); 1.97(0.94)		13.3 (3.2); 11.9 (1.5)	10.7(1.1); 10.6(0.95)		
Elif Guler Kazanci	2019	Retrospective study	sPDA group, n=169；nsPDA group, n=312	1 day	219 (172–265); 229 (176–280)	1648 (1329–2142); 1852 (1503－2237)				0.034 (0.026–0.045); 0.034 (0.026–0.047)	13±2.8; 13.3 ±2.2	7.6±0.9; 8.1±1		
Temel MT	2017	Retrospective study	Case group, n=47；Control group, n=50	3 day	220.47±72.50; 254.58±83.19						11.27±2.14; 11.15±1.77	10.00±0.89; 10.02±0.94	0.22±0.07; 0.25±0.08	
Junyan Zhong	2021	Retrospective study	PDA closure group, n=70；PDA open group, n=59	1 day	212 (170－248); 183 (144－232)						16.9 (16.6－17.2); 17.0 (16.7－17.3)	9.8 (9.3－10.9); 10.3 (9.4－10.9)	21.4 %(16.9－23.4); 19.1% (14.8－23.9)	

Ozgur Olukman	2016	Retrospective study	Case group, n=208；Control group, n=616	3-4 day	186 (108–257); 198 (119–314)				1506 (720–2210); 1584 (768–2731)		12.2±1.2; 11.8±1.7	8.1±0.5; 8.0±0.7		
Tai-Xiang Liu	2023	Retrospective study	Succeed group, n=83；Failed group, n=99	3 day	208 ± 92; 183 ± 86									
Birol Karabulut	2019	Retrospective study	sPDA Case group, n=71；nsPDAControl group, n=77	1 day;2 day;3 day;7 day	24h(211 ± 43,257 ± 64); 48h(171 ± 35,255 ± 47); 72h(159 ± 23,199 ± 51); 7d(262 ± 66,311 ± 78)						24h(18 ± 2.2,17.7 ± 1.8); 48h(18 ± 2.29,18.1 ± 1.98); 72h(18.6 ± 2.7,17.3 ± 2.1); 7d(18.4 ± 1.4,18.9 ± 1.8)	24h(8.7 ± 1.7,8.3 ± 2.3); 48h(9.7 ± 1.9,8 ± 1.4); 72h(9.8 ± 2.4,8.8 ± 2.9); 7d(7.6 ± 2.3,8.3 ± 2.4)		
Dilek Kahvecioglu	2017	Retrospective study	Open PDA group, n=24；Closed PDA group, n=36	3 day	181 (64–277); 189.5(52-58)	1448 (467–2246); 1473(530-4872)						8.2 (7–9.8; 8 (7-10.2)		

Yanjun Z	2020	Case-control study	Case group, n=124；Control group, n=124	7 day	214.00(165.00-264.25）; 240.00(195.00-280.50)						16.70(16.48-17.00); 16.70(16.40-16.90)	9.25(8.90-9.83); 9.20(8.80-9.60)	19.65%(15.78-24.10); 21.80%(18.23-25.48)	
Baoli Z	2023	Case-control study	Case group, n=54；Control group, n=54	1 day	185.56±29.83; 204.74±30.26						13.35±2.51; 13.77±2.70	10.24±1.93; 12.53±2.05	0.17±0.03; 0.26±0.05	
Yuan W	2021	Case-control study	Case group, n=80；Control group, n=80	1 day	196.23±37.18; 196.23±37.18						13.27±1.499 ; 15.06±1.71	9.13±1.56; 10.04±1.53	0.12±0.04; 0.23±0.03	30.20±3.61 ; 29.13±4.90
Jia C	2012	Case-control study	Case group, n=266；Control group, n=250	1 day	212±6; 229±79									
Tiantian L	2015	Case-control study	Case group, n=136；Control group, n=136	1 day	211.10±62.69; 229.14±65.55									
Senjing C	2017	Case-control study	Case group, n=33；Control group, n=33	1 day	190.42±63.47 ; 229.00±67.27						11.50-14.25; 10.95-13.50	10.50±1.06; 10.11±0.88		

Jingye Y	2023	Case-control study	Case group, n=57； Control group, n=35	3 day	222.36±7.51; 245.76±10 34						12.69±0.24; 12.54±0.35	10.60±0.09; 10.48±0.10	0.23±0.01; 0.26±0.01	29.82±0.70 ; 28.67±0.85
Chunxia L	2018	Case-control study	Case group, n=68； Control group, n=60	1 day	192.68±32.4; 207. 49±28. 96						12.26±1.57; 12.09±2.09	9. 36±0. 75; 11. 13±0. 63	0.14±0.03; 0.26±0.05	30.75±7.48 ; 28.69±5.36
Yali Z	2019	Case-control study	Case group, n=26； Control group, n=35 group, n=	1 day	222.88 ± 54.14; 224.51 ± 62.69								3.64 ng/ml (1.29,9.30); 3.44 ng/ml (1. 10,8.51)	
Jingfeng D	2016	Case-control study	Case group, n=136； Control group, n=136	1 day	211±63; 229±66									
Yi R	2021	Case-control study	Case group, n=24； Control group, n=48 group, n=	4-5 day	191±82; 221±44						0.13±0.02; 0.13±0.02	10.3±2.4; 9.2±2.0	0.0021±0.000 9; 0.0028±0.000 9	0.25±0.07; 0.22±0.07

Yi R	2019	Case-control study	sPDA group, n=67；nsPDA group, n=106；nPDA group, n=587	1 day	sPDA group 238 ±65; nsPDA group 262 ±68; nPDA group 286 ±85						sPDA group 11.3 ±1.1；nsPDA group 11.2 ±1.2；nPDA group 11.3 ±1.2	sPDA group 10.2 ±1.2；nsPDA group 10.3 ±1.0；nPDA group 10.2 ±0.8	sPDA group 0.24 ±0.07；nsPDA group 0.27 ±0.06；nPDA group 0.28 ±0.09	sPDA group 25.66±6.5；nsPDA group 25.66±5.3；nPDA group 25.3 ±6.3

Note: Case group was PDA group, control group was nPDA group, sPDA was hemodynamic PDA, nsPDA was non-hemodynamic PDA. platelet count (PLT) ×109/mm3; Platelet mass fL/nL; Platelet count nadir (×109/l); red cell distribution width--to--platelet ratio (RPR); Red cell distribution width--to--platelet ratio (RPR); platelet-derived, PDGF (pg/ml); mean platelet volume to platelet ratio (MPR); platelet distribution width (PDW)%; mean platelet volume (MPV)fL; plateletcrit (PCT); platelet-larger cell ratio (P-LCR).
