# Supplementary material for: Relationship between patent ductus arteriosus and platelet indices in newborn: a systematic review and meta-analysis
Source: Front Pediatr. 2025 Mar 21;13:1455183. doi: 10.3389/fped.2025.1455183 (PMC11968669; doi:10.3389/fped.2025.1455183)
Supplement: Supplementary file 2 [file Datasheet2.pdf]

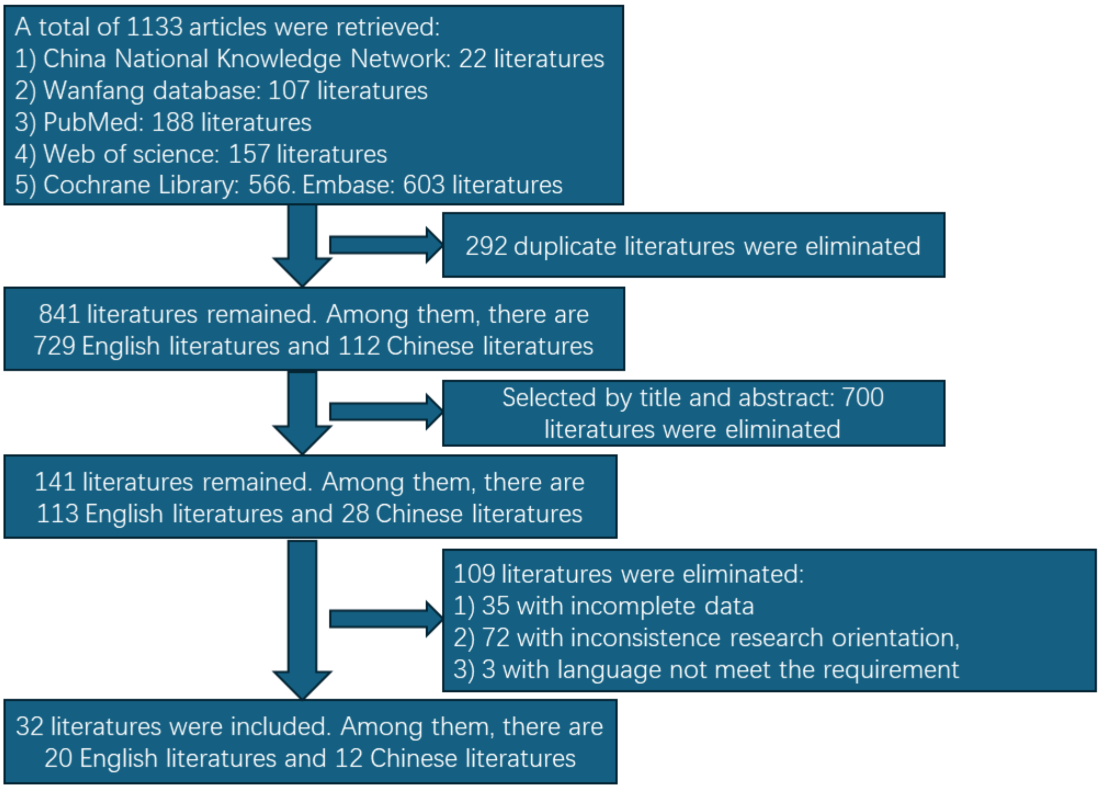

Figure S1 The inclusion and exclusion flowchart for the literatures.

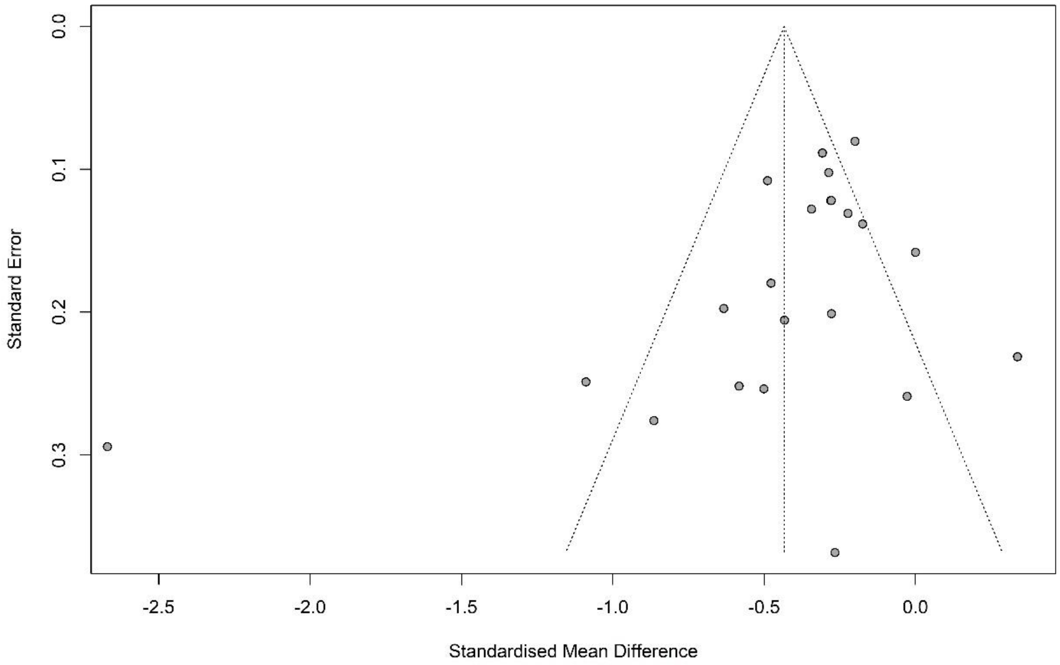

Figure S2A Funnel plot for the comparison of PLT between newborns with PDA and non-PDA

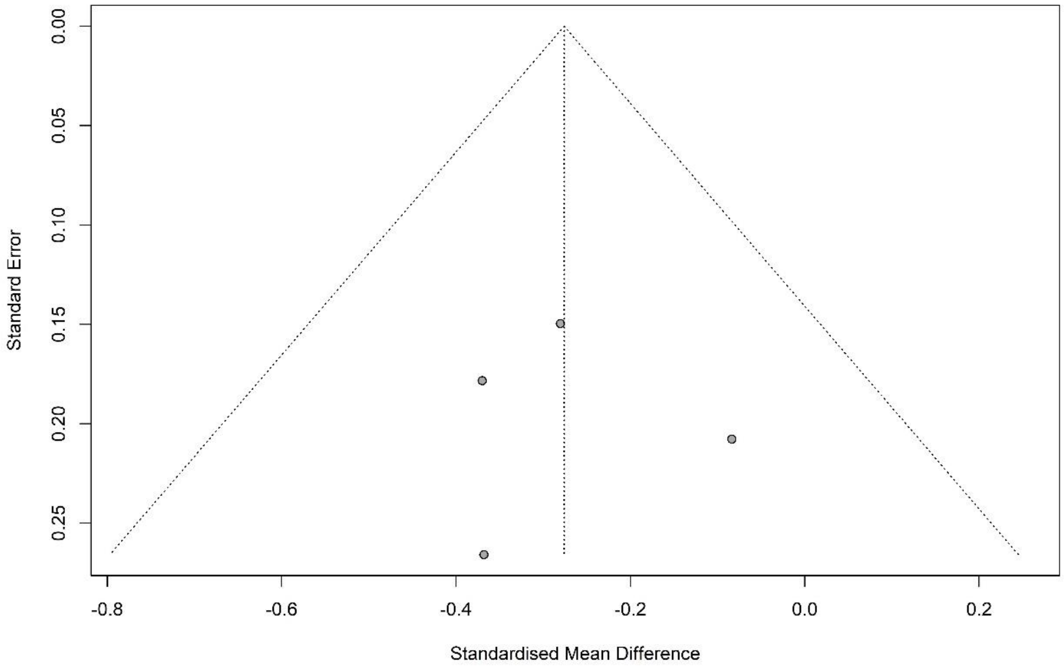

Figure S2B Forest plot for the comparison of PLT between newborns with PDA and PDA closure

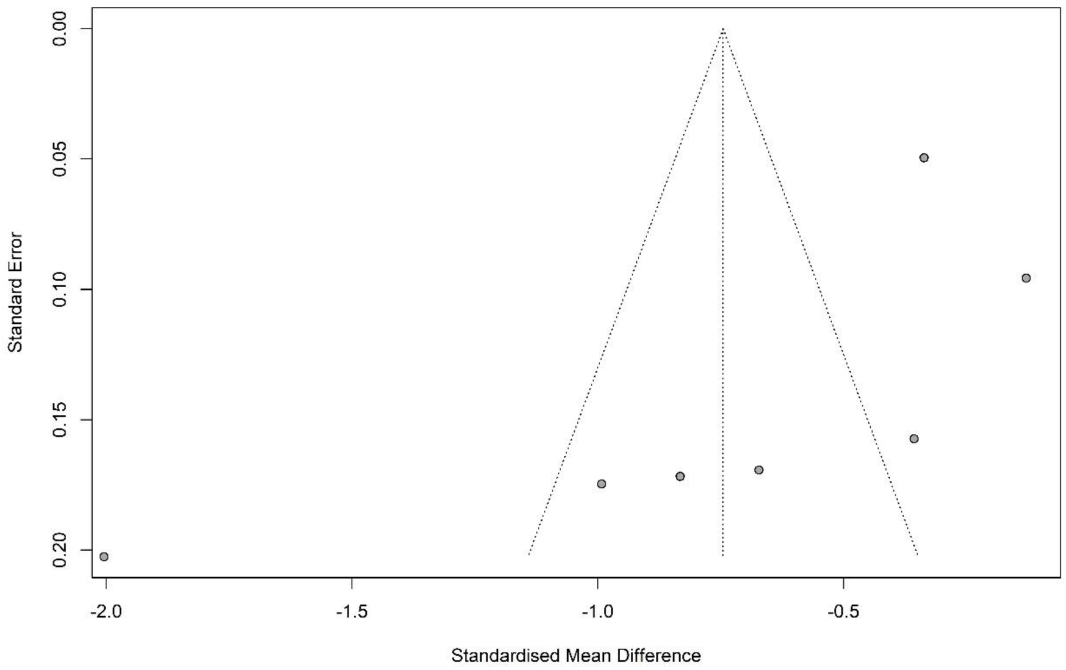

Figure S2C Funnel plot for the comparison of PLT between newborns with sPDA and non-sPDA

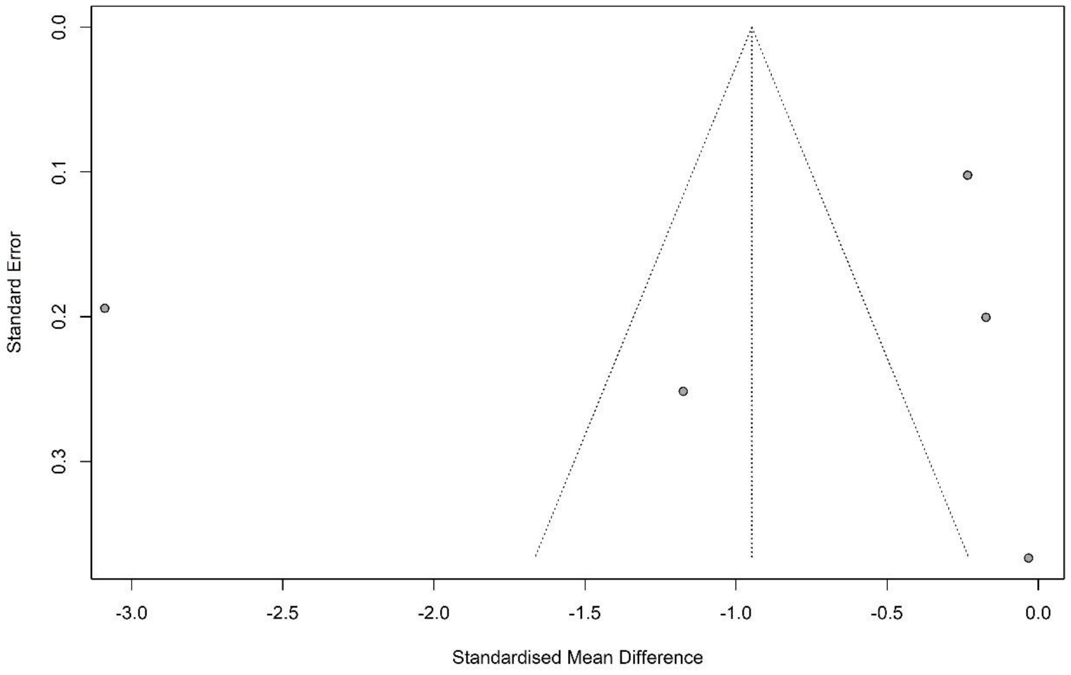

Figure S3 Funnel plot for the comparison of platelet quality between newborns with PDA and non-PDA

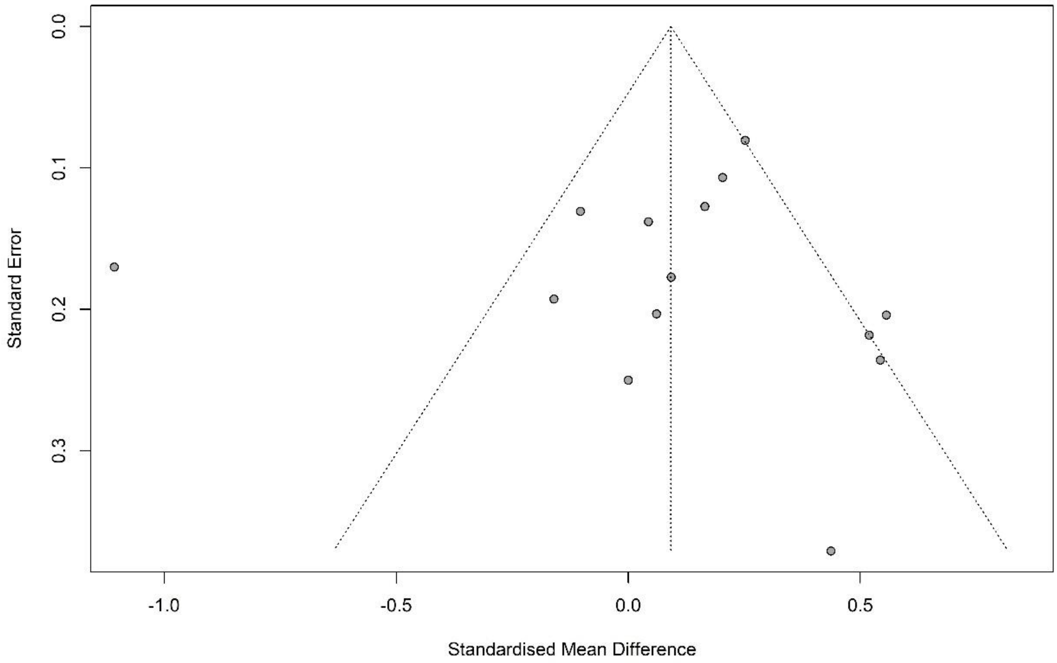

Figure S4A Funel plot for Comparison of PDW between newborns with PDA and non-PDA

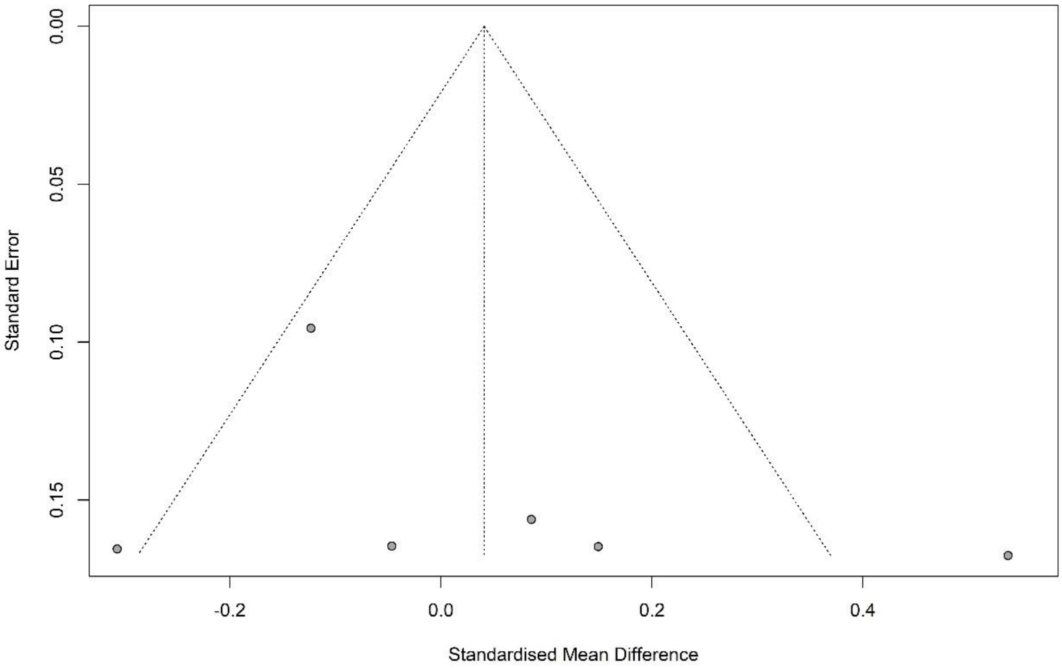

Figure S4B Funel plot for Comparison of PDW between newborns with sPDA and non- sPDA

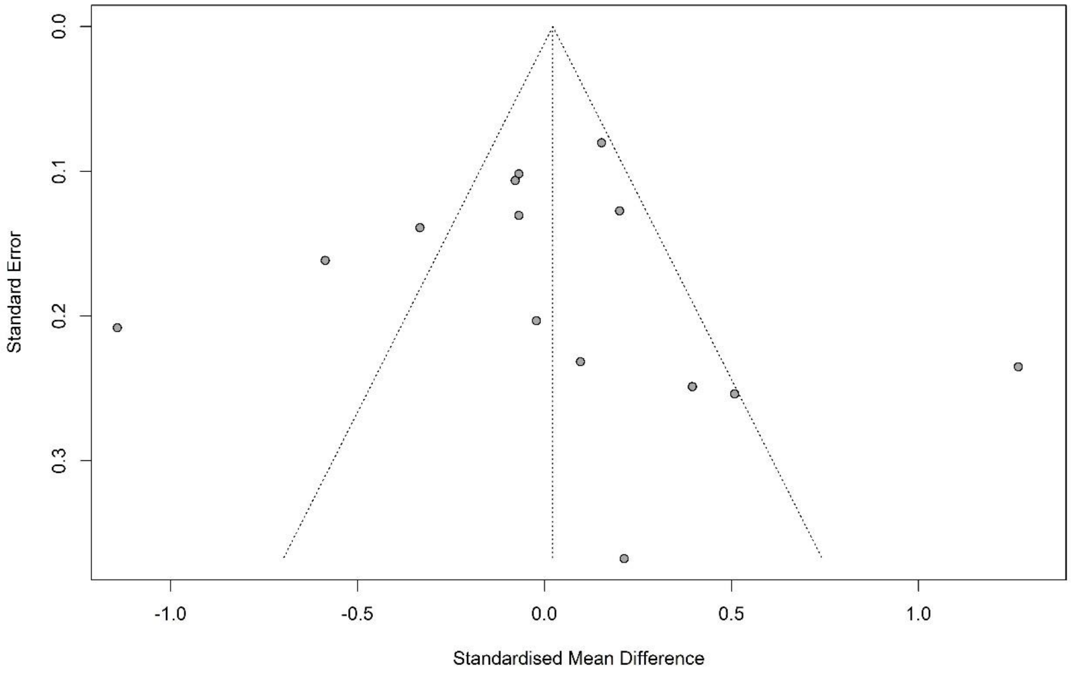

Figure S5A Funnel plot for comparison of MPV between newborns with PDA and non-PDA

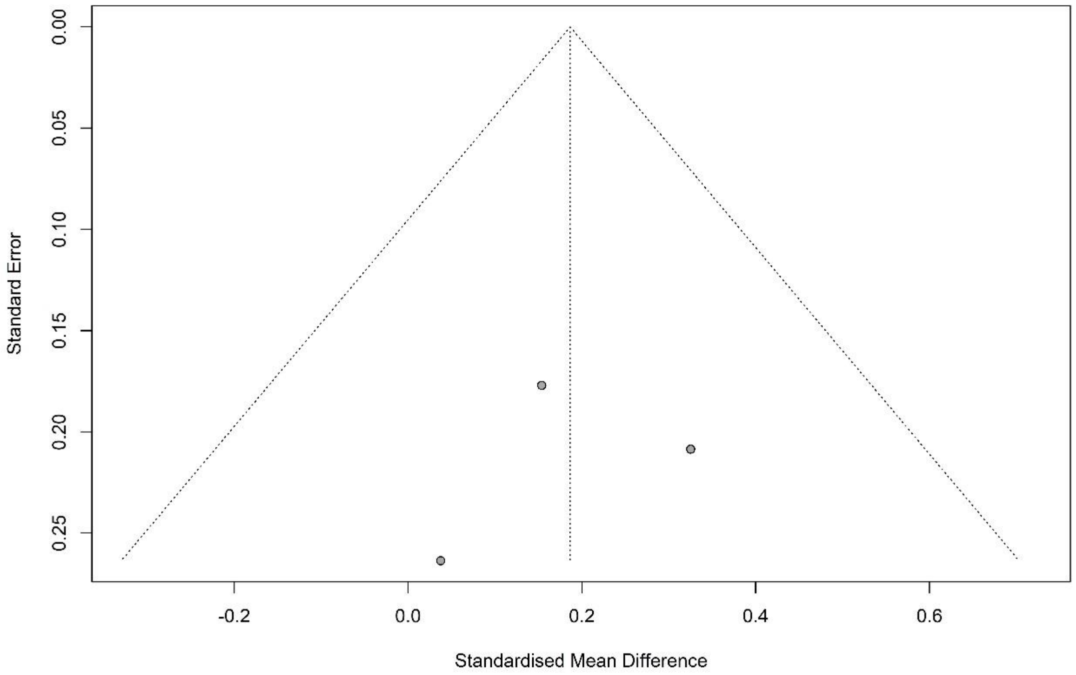

Figure S5B Funnel plot for comparison of MPV between newborns with PDA and PDA closure

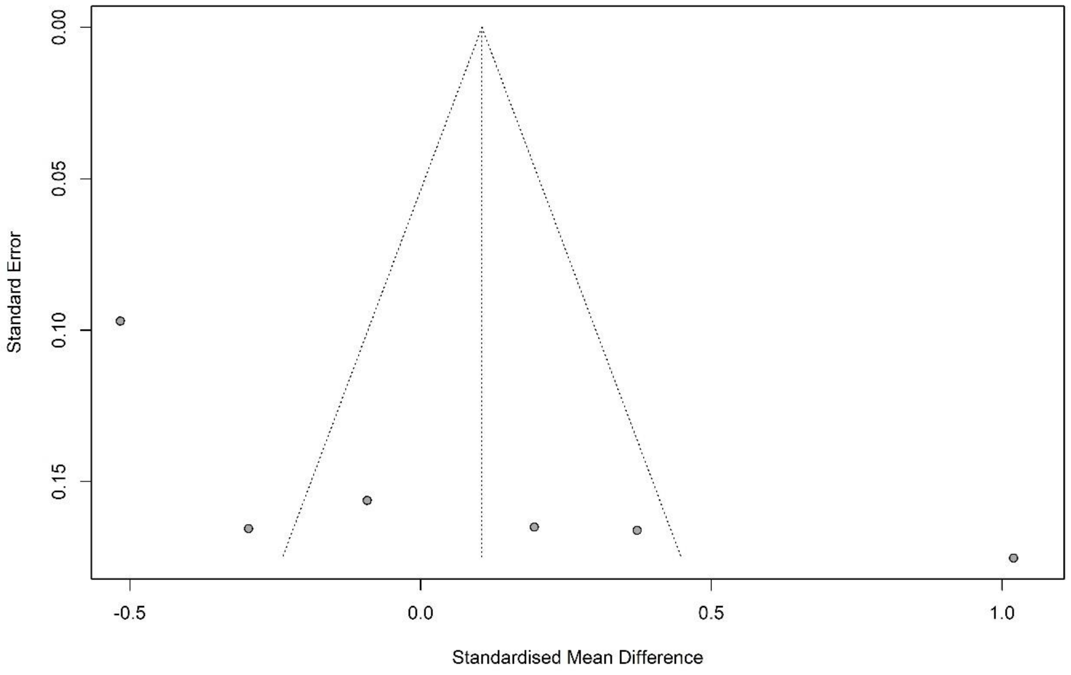

Figure S5C Funnel plot for comparison of MPV between newborns with sPDA and non-sPDA

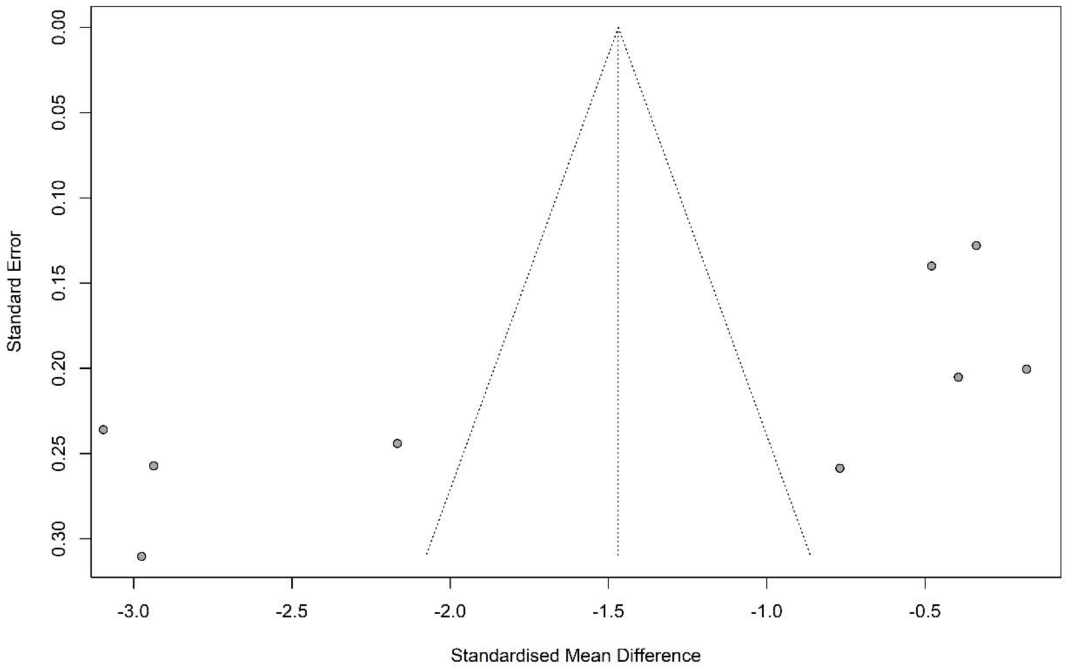

Figure S6 Funnel plot for comparison of PCT between newborns with PDA and non-PDA

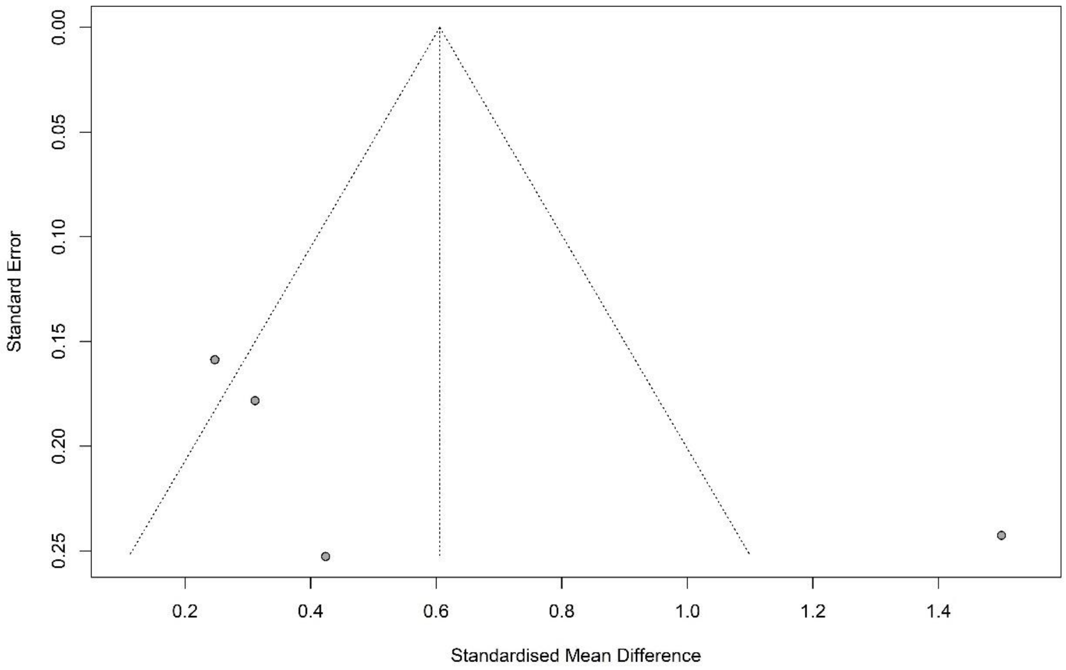

Figure S7 Funnel plot for comparison of P-LCR between newborns with PDA and non-PDA
